# Practical Notes and Formulæ

**Published:** 1884-06-20

**Authors:** 


### PRACTICAL NOTES AND FORMULÆ.

**Chronic Enlargement of Spleen.**—

℞ Podophyllin.............................grs. iv,

Iodide potash.............................ʒ ij,

Fld. ext. stillingia.............................ʒ iv,

Fld. ext. phytolacca.............................ʒ iv,

Syrup rhei. pot. comp.............................℥ iij.

Teaspoonful in water one, two or three times a day after meals, using freely comp. tinct. iodine externally.

℞ Tinct. cinchona comp.............................℥ viij,

Ammon mur.............................℥ j,

Aqua menth. pip.............................℥ xij.

Teaspoonful three times a day before meals.—*Med. World.*

℞ Quinine sulph.............................ʒ ij,

Ammon iod.............................ʒ ijss,

Liq. potass. arsen.............................ʒ ss,

Glycerine.............................℥ iv,

Dissolve and take a teaspoonful three times a day.

**Hair Tonic.**—Dr. Gross suggests the following formula:

℞ Tinct. cantharidis.............................ʒ jss,

Tinct. capsici.............................gtt. xx,

Glycerini.............................ʒ ss,

Aquæ cologniensis.............................q. s. ad ℥ vj.

M.

**An Anodyne Mixture without Opium.**—Dr. A. P. Meylter writes to the Medical Record that it has given him no little anxiety to find a suitable anodyne for patients cured of the opium habit. In attacks of intestinal colic or neuralgia, to which they are subject, any preparation containing morphia is necessarily excluded, since it would at once bring back the old habit. The following formula is the result of various experiments, and has been used with good results in his cases:

℞ Chloroform.............................100
Ether sulphur spts.............................025
Tinct. cannabis.............................175
Acid. hydrocyan. dil.............................030
Hyosciamia.............................q. s.
Ol. menth. piperit.............................003
Tinct. capsici.............................003
Alcohol, 95 per cent.............................350
Glycerine.............................ad 1.000

Dose 10-30*m.* —*Ibed.*

**For Dysenteric Diarrhœa.**—In a case of diarrhœa with dysenteric tendency, after a dose of castor oil or epsom salts, give the following :

℞ Sulphate morphine........................gr. ij,
Tannin..............................gr. xx,
Ipecac..............................gr. x,
Loaf sugar..........................gr. xxx.

M. Make powders No. x.

S. Give one every two to four hours to an adult until the discharges are restrained, and then one after each evacuation until sufficiently controlled.

**For Dysentery,**—

℞ Epsom salts..........................½ ounce,

Water..............................½ pint,

Muriate morphine.....................1 gr.

M.

S. Two teaspoonfuls every hour to an adult until discharges cease, or are changed, and then lengthen the interval. If fever be present, add half drop tincture aconite to each dose. This remedy with dose reduced may be used also with children.

**For Bilious Dysentery.**—

℞ Calomel..............................gr. x,
Dovers powder........................grs. v.

M.

Give at a dose and follow it with the following :

℞ Epsom salts.........................ij drachms,
Water..............................6 ounces.

M.

S. Tablespoonful every hour until two or three bilious evacuations are secured. Then arrest with 8 to 10 grains of Dovers powder, applying large mustard poultice to abdomen. If fever be present give aconite.

**Asthma.**—

℞ Tinct. lobelia.........................℥ v,

Ammon. iodide.........................ʒ ij,

Ammon. bromide........................ʒ iij,

Syr. tolu.............................℥ iij.

M.

S. Teaspoonful every two, three or four hours.

—*Fothergill.*

**Prevention of Pitting in Small-pox.**—We take the following from the Polyclynic. Schwimmer recommends the local application of carbolic acid and thymol. He prescribes as follows :

℞ Acid carbol.................................ʒ j,

Ol. oliv......................................ʒ viij,

Cretæ. prep.................................℥ iss.

M. Or,

℞ Thymol......................................ʒ j,

Ol. lini.......................................ʒ viij,

Cret. prep....................................℥ iss.

M.

—*Med. Digest.*

**Scrotal Puritus.**—Scrotal puritus can be cured in a few days by rubbing on the affected parts the following ointment night and morning, and washing them with strong soap suds every other day :

℞ Vaseline or cosmoline..........................℥ i,

Lac sulphur.....................................ʒiij,

Oil burgamot.....................................ʒi,

M.

*I. H. Curnalla, M. D., in Brief.*

℞ Quinine.........................................ʒ i,

Adeps............................................q.s.

M.

Apply to scrotum three times a day, and bathe it in salt water every night before going to bed.—*C. Hoover, in Brief.*

℞ Saturated solution of salicylic acid, applied three times a day to the scrotum.—*Lancet.*

℞ Balsam peru, apply to scrotum twice a day.

℞ Acetate of lead...............................ʒ i,

Acetic acid......................................mx,

Glycerine........................................℥ iss,

Water............................................℥ii.

M. Apply to scrotum.

—*E. C. Med. Jour.*

**Treatment of Pruritus Ani and Ascarides.**—A writer in the Medical and Surgical Reporter, says :

The causes of pruritus ani are either general or local. Sometimes the cause is entirely local; but most frequently it is both general and local. These causes should be, if possible, ascertained. The patient should be instructed to wash his anus well with a cloth wet with cold water immediately after each action of the bowels—this cleanliness is an exceedingly important agent in the treatment of the disease—and after so washing and drying himself, to bathe his anus with the following :

℞ Hyposulphite of soda............................ʒ j,

Carbolic acid.................................grs. x,

Glycerine.....................................ʒ j,

Aqua distil...................................℥ vij.

M.

For a lotion.

Whenever the itching returns the application of the lotion is to be repeated and continued until the disease is arrested. Sometimes a good result is obtained by dusting over the anus the following :

℞ Iodoform......................................ʒ j,

Tannic acid...................................grs. x.

M.

For a powder. Use two or three times a day.

I have found an injection of two ounces of infusion of pulv. aloes (ʒj. to the pint of boiling water), night and morning, with a teaspoonful or two, twice or thrice a day, for three or four days, of the following mixture, to be most efficient in the removal of ascarides :

R Santonin......................................℈ j,

Pulv. jalap...................................℈ ij,

Fld. ext. spigelia.............................℥ j,

Fld. ext. senna................................℥ ss,

Syrup sarsaparilla comp........................℥ jss.

M. Shake well.

S. Dose, one to two teaspoonfuls three times a day, followed in a few days, if indicated, with suitable doses of mur. tinct. of iron in an infusion of quassi.

**Asthma.**—Time out of mind almost, iodide of potassium has been a standard remedy in the treatment of asthma. It succeeds more frequently than any other remedy. Combining the iodide of potassium with the iodide of ammonium in equal quantities, we think, gives better results—that is, affords relief quicker, and more permanent in effect. We order :

℞ Iodide potassium, }

Iodide ammonium, } ..........................aa ʒ i,

Syrup wild cherry..............................℥ iv.

M.

S. Teaspoonful every two hours until relief is obtained, then at longer intervals. When the patient complains of a sense of difficulty in breathing out the air from the lungs—a complaint some patients make, and who are pale, and very distressed about this symptom—we add the gelseminum in five-drop doses, which seldom fails to bring relief, and that, too, promptly. Lobelia affords relief to those who complain of constriction across the chest, and have a rather livid countenance and bluish color of the mucous membranes of the lips and mouth. This, added to the iodide, answers in these cases finely, and we prefer the tincture of lobelia seed to the herb.—*E. C. Med. Jour.*

